# Posterior to anterior malleolar extended lateral approach to the ankle (PAMELA): early results of a novel approach

**DOI:** 10.1007/s00402-022-04360-1

**Published:** 2022-02-03

**Authors:** Anne Kummer, Xavier Crevoisier

**Affiliations:** Department of Orthopedics and Traumatology, Lausanne University Hospital (CHUV), and University of Lausanne (UNIL), Pierre-Decker 4, 1011 Lausanne, Switzerland

**Keywords:** Ankle fracture, Trimalleolar fracture, Posterior tibial malleolus, Extended approach to the ankle, Chaput tubercle

## Abstract

**Introduction:**

In a previous cadaveric study, we described the Posterior to Anterior Malleolar Extended Lateral Approach (PAMELA) to address complex ankle fractures. It was demonstrated to provide optimal exposure of the posterior and lateral malleoli, and of the anterolateral portion of the ankle through a single incision. The aim of this study is to report the clinical results of this novel approach.

**Methods:**

Between January 2019 and January 2021, all patients presenting with a complex ankle fracture in our institution were assessed by CT scan. Indication to use the PAMELA was determined by the fracture pattern, according to our previous guidelines, including combination of complex lateral and displaced posterior malleolar fractures, associated in most cases with anterolateral fracture avulsion. The approach was performed according to the steps previously described. Intraoperative evaluation included quality of exposure, ease of performing the osteosynthesis, and any complication encountered. The postoperative course was assessed for wound healing, quality of reduction, and the occurrence of any complication.

**Results:**

The PAMELA was performed in 20 patients (aged 17–73). The most common combination of fractures was a comminuted lateral malleolus fracture associated with a displaced fracture of the posterior malleolus and a Wagstaffe-Le Fort or Chaput avulsion. We encountered no intraoperative complication. X-rays showed anatomical reduction in all cases. Postoperative complications included three delayed wound healing resolved with local treatment and one sural nerve traction injury.

**Conclusions:**

The main potential concern regarding this novel approach was the healing of the flap. Our results reject this concern and are in line with wound healing complications reported following surgical treatment of ankle fractures. This study confirms the safe in vivo feasibility of the PAMELA and opens a new perspective in the optimal management of complex fractures of the ankle. A larger prospective clinical study is ongoing in our institution.

## Introduction

The prognosis of ankle fractures depends largely on obtaining an anatomical reduction [1], and particular attention to the posterior malleolus is currently recognized to achieve this goal [2, 3]. Furthermore, recent literature highlights the importance of considering and anatomically restoring the anterolateral portion of the ankle, such as the anterior syndesmosis [4, 5] or a Chaput or Wagstaffe-Le Fort fracture avulsion [6–8]. In case of complex ankle fracture, to restore the anatomical relationship and congruency of the anterolateral portion of the ankle is, thus, of great importance. Moreover, the visualization of the anterior tibio-talo-fibular junction provides substantial information to ensure the anatomical reduction of comminuted fractures, when diaphyseal and metaphyseal bony landmarks are less reliable [9].

In a previous cadaveric study, we described the posterior to anterior malleolar extended lateral approach (PAMELA) [9]. This new approach allows optimal exposure of the posterior and lateral malleoli together with exposure of the anterolateral portion of the ankle through a single incision. The main potential concern regarding this extensive approach was the healing of the cutaneous flap. Therefore, after the cadaveric study, the mandatory next step was the clinical application of this approach to prove the clinical feasibility and the safety of the PAMELA to treat complex ankle fractures. The aim of this study is to report the early results of this novel approach.

## Methods

### Materials

This investigational protocol was conducted with the approval of the institution's ethics committee (Commission cantonale d'éthique de la recherche sur l'être humain CER-VD, ID 2020-02862).

Between January 2019 and January 2021, all patients presenting with a complex ankle fracture in our institution were assessed by conventional X-rays and a CT scan. The posterior malleolus fracture was classified according to Haraguchi [10] and Bartonìček [11]. Indication to use the PAMELA was determined by the fracture pattern, according to our previous guidelines (Table 1) [9] and validated by the senior author. All patients who sustained an ankle fracture pattern leading to the indication to perform a PAMELA were retrospectively included in the present study. Of note, although the patients were retrospectively included in the analysis, the data were prospectively collected for each patient.

**Table 1:** Indications for the posterior to anterior malleolar extended lateral approach (PAMELA)

**Fracture**
**1.** Simple fracture of the lateral malleolus
**2.** Comminuted fracture of the lateral malleolus
**3.** Displaced fracture of the posterior malleolus*
**4.** Chaput tubercle avulsion fracture
**5.** Wagstaffe-Le Fort avulsion fracture
**6.** Fracture of the anterolateral talar body
**7.** Fracture of the posterior talar body
**Combinations of fractures leading to indication for the PAMELA**
**A.** 1 + 3 + 4 or + 5 or + 6 (or + 7)
**B.** 2 + 3**
**C.** 2 + 3 + 4 or + 5 or + 6 (or + 7)
**D.** 2 + 7

We decided to write “(or + 7)” in the combination section because a posterior talar body fracture is rarely present if the posterior malleolus is already fractured

*Fractures of the posterior malleolus types 1 and 2 according to Haraguchi, [10] types 2, 3 and 4 according to Bartoníček [11] included

**Some combinations B can be treated using a standard posterolateral approach. The PAMELA is dedicated to combination B with complex comminution of the fibula resulting in loss of sufficient bony landmarks, thus necessitating to visualize the anterior tibio-talo-fibular junction to confirm anatomical position of the distal fibula

### Surgical procedure and postoperative care

For each patient, the following standardized procedure was performed.

The patient was installed in lateral decubitus position, or, in case of associated medial malleolus fracture, in 3/4 supine position on a vacuum mattress to allow access to the medial side of the ankle by tilting the operating table on the injured side of the patient. An antibioprophylaxis of cefuroxime 1.5 g was administered and a tourniquet was applied.

**Fig. 1:**
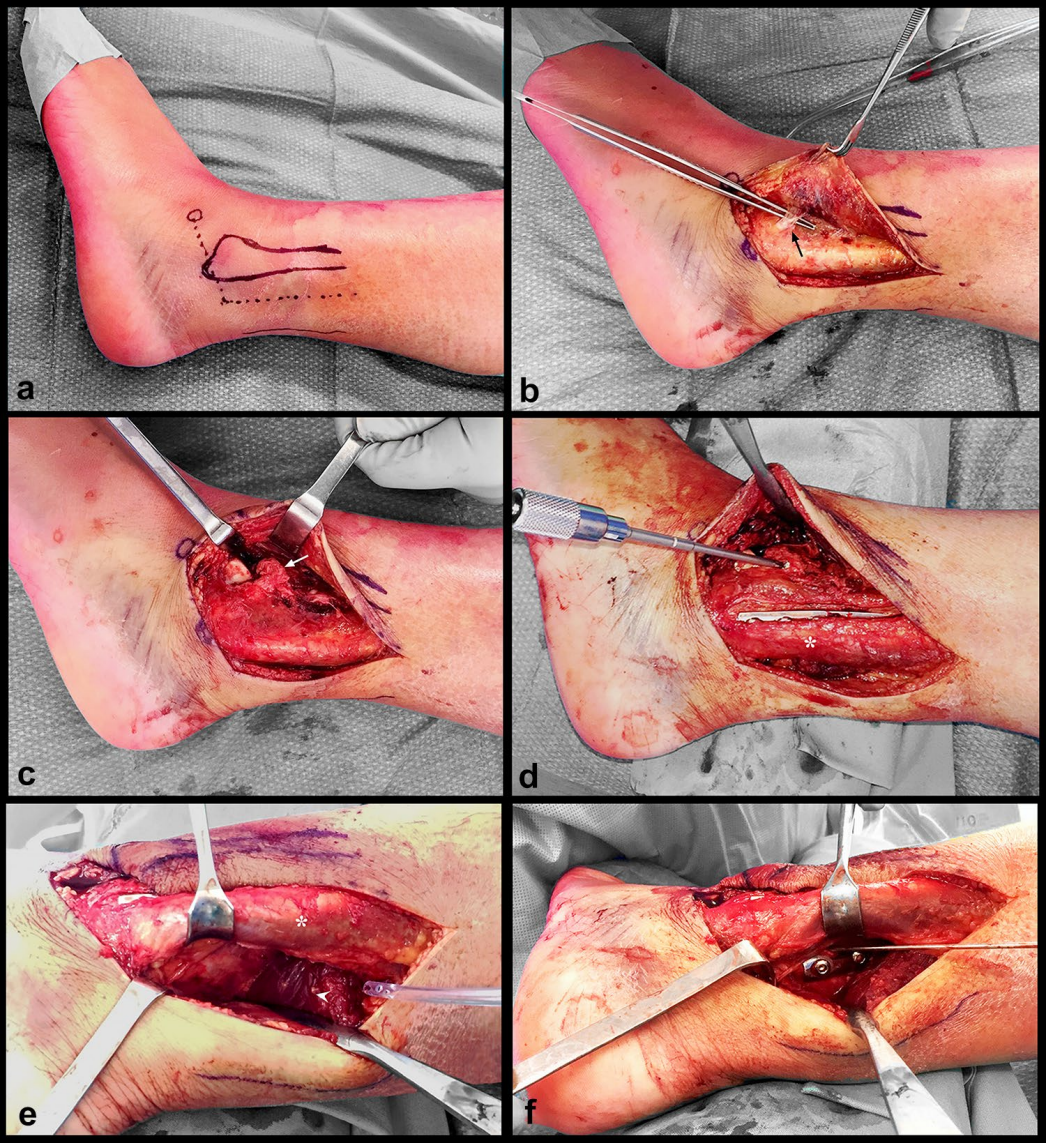
**a** Landmarks (lateral malleolus, sinus tarsi) and incision in dotted line. **b** Elevation of the thick anterior flap, with exposition of the inferior extensor retinaculum (black arrow) which is partially incised to reach the anterior talofibular ligament. **c** The avulsion fracture of the Chaput tubercle is exposed (white arrow), as well as the superolateral talar dome. **d** Osteosynthesis of the Chaput tubercle performed with one 3.5 cortical screw and fixation of the lateral malleolus with a plate positioned by retracting the peroneal tendons (*) posteriorly. **e** Exposition of the posterior malleolus through the interval between the peroneal tendons (*) and the flexor hallucis longus (arrow head). **f** Direct access for osteosynthesis of the posterior malleolus

The approach was performed according to the following steps, as described in our cadaveric study [9]:

1. Standardized L-shaped incision (Fig. 1a): the longitudinal portion is located at the junction between the anterior third and the two posterior thirds of the interval between the posterior edge of the fibula and the lateral edge of the Achilles tendon. It is carried out from the level of the tip of the fibula and extends proximally for a distance of about 10 cm. The distal part of the incision continues obliquely, forming an angle of 110° with the longitudinal portion, on a straight line defined by the tip of the lateral malleolus and the sinus tarsi.
2. The deep incision is carried down directly until the peroneal fascia. Then, a full-thickness flap is elevated anteriorly, exposing the lateral malleolus. The flap is developed distally, exposing the inferior extensor retinaculum (Fig. 1b) which is partially incised to reach the anterior talofibular ligament. Then, arthrotomy is performed in the interval between the anterior talofibular and the anterior tibiofibular ligaments to expose the anterior aspect of the tibio-talo-fibular junction and the anterior superolateral margin of the talar dome (Fig. 1c, d).
3. The fibula is exposed by retracting the peroneal tendons medially.
4. The posterior malleolus is exposed by developing the interval between the flexor hallucis longus (FHL) and the peroneal tendons, which are retracted laterally (Fig. 1e). To reach this interval, the crural fascia is incised longitudinally in line with the skin incision, which allows to protect the sural nerve and the lesser saphenous vein when retracting the fascia posteriorly, as their courses are located slightly posteriorly, halfway between the lateral malleolus and the Achilles tendon, in the subcutaneous tissue (epifascial). Nevertheless, anatomical variations of the sural nerve are frequent and care must always be taken before incising the fascia. The approach allows to expose 80% of the width of the posterior tibial portion.

Once the approach was done, reduction and fixation of the different fractures were performed with standard DCP plates and 3.5/2.7 mm screws, or screws alone (Chaput tubercle), or osteosuture (anteroinferior tibiofibular ligament), depending on the pattern and the location of the fractures. Usually, we performed the reduction and fixation of the medial malleolus first if it was fractured, then we addressed the posterior malleolus and the lateral malleolus, and finally the Chaput tubercle if needed.

Closure of the wound begun with the inferior extensor retinaculum using 2–0 Vicryl. The crural fascia was sutured with interrupted stiches of 0 Vicryl. The subcutaneous tissue was closed with 2–0 Vicryl. The skin was closed with Donati stiches, or uninterrupted suture using 3–0 nylon. Then, the ankle was immobilized in a splint in neutral position.

Postoperative care consisted of elevation of the limb and ice application during the first 24–48 h. Then, a removable cast was done together with the first change of the wound dressing and the patient begun to walk with partial weight bearing for 6 weeks. The wound was checked once a week until removal of the stiches at 3 weeks. At 6 weeks, the cast was removed and the patient was allowed to walk with weight bearing as tolerated.

Control X-rays were done at 48 h (mortise and lateral ankle views), 6 weeks and 3 months (weight-bearing mortise and lateral ankle views).

### Assessments

Demographic data, relevant comorbidities at risk of influencing wound healing, and mechanism of injury were recorded. Intraoperative evaluation included the quality of exposure, the ease of performing the osteosynthesis, and any difficulty or complication encountered. The postoperative course was assessed for wound healing, for quality of reduction on standard X-rays according to Burwell and Charnley [12], and for the occurrence of any complication.

### Statistical analysis

Data were collected and analyzed using REDCap® and IBM SPSS Statistics. Descriptive statistics are reported as median (interquartile range) or mean (± standard deviation) as appropriate for continuous variables (e.g. age, operative duration), and absolute or relative frequencies for categorical variables (e.g. combinations of fracture (A/B/C/D), intraoperative and postoperative complications).

## Results

Between January 2019 and January 2021, the PAMELA was performed in 20 patients, 11 women and 9 men. Demographics are shown in Table 2. The median follow-up was 21 weeks (12–72 weeks).

**Table 2:** Demographics and preoperative characteristics

	*n* = 20
Mean age (range, SD)	46 (17–73, 17)
Mean BMI (kg/m^2^), (SD)	26.6 (4.1)
Relevant comorbidities related to wound healing, *n* (%)	
Smoking	1 (5)
Diabetes mellitus	2 (10)
Dermatologic disease	1 (5)
Oral anticoagulant	1 (5)
Immunosuppression	0
Venous insufficiency	0
ASA grade, *n* (%)	
1	5 (25)
2	13 (65)
3	2 (10)
4	0
Side, L/R	9/11
Mechanism of injury	
Low kinetics	20/20
Open fracture	0/20
Mean time from injury to surgery (days), (range, SD)	9.5 (0–13, 2.95)

Combinations of fractures leading to this approach was C for nine patients (45%), A for six patients (30%) and B for five patients (25%). The configuration of the posterior malleolus fractures (Table 3) according to Haraguchi [10] was a type 1 (posterolateral oblique) in 13 patients and a type 2 (transverse medial-extension) in 7 patients. According to Bartonìček [11], there were 12 type 2 (intraincisural posterolateral fragment), 7 type 3 (intraincisural two-part fragment involving the medial malleolus) and 1 type 4 (intraincisural large triangular fragment) fractures.

**Table 3:** Posterior malleolus fractures configuration in the study population, compared to relative frequencies according to Haraguchi et al. [10] and Bartonìček et al. [11]

Haraguchi classification, type	*n* (%)	Relative frequency in the original article (%)	Bartonìček classification, type	*n* (%)	Relative frequency in the original article(%)
1	13 (65)	67	1	0	8
2	7 (35)	19	2	12 (60)	52
3	0	14	3	7 (35)	28
			4	1 (5%)	9
			5	0	3

The mean time between injury and surgery was 9.5 days (range 0–13, SD 2.95). The mean surgery duration was 176 min (range 94–311, SD 54). We encountered no complication during the procedure. Quality of exposure and access for osteosynthesis were optimal in all cases. Postoperative X-rays showed anatomical reduction in all cases.

**Fig. 2:**
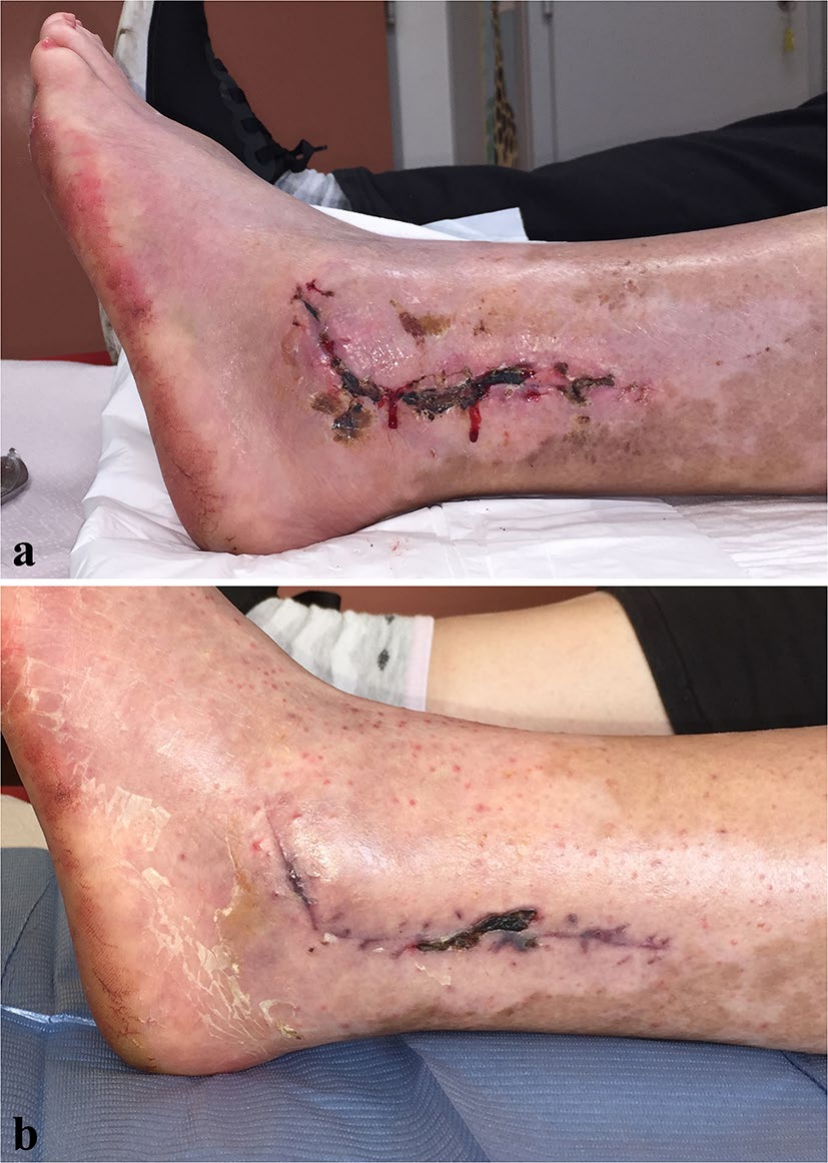
An example of wound complication following the PAMELA, at 22 (**a**) and 42 (**b**) postoperative days

Postoperative complications included three delayed wound healing resolved with local treatment and one sural nerve traction injury. Among the three patients presenting delayed wound healing, one patient was diabetic. These three wound complications consisted of slight dehiscence and superficial necrosis located along the longitudinal portion of the incision (Fig. 2), without extension at the level of the skin flap, and healing was acquired with wound dressing and local treatment between 6 and 8 postoperative weeks, without further complications. The patient presenting the sural nerve traction injury complained of hypoesthesia on the lateral side of the foot, which was resolved at the follow-up at 1 year.

## Case presentation

**Fig. 3:**
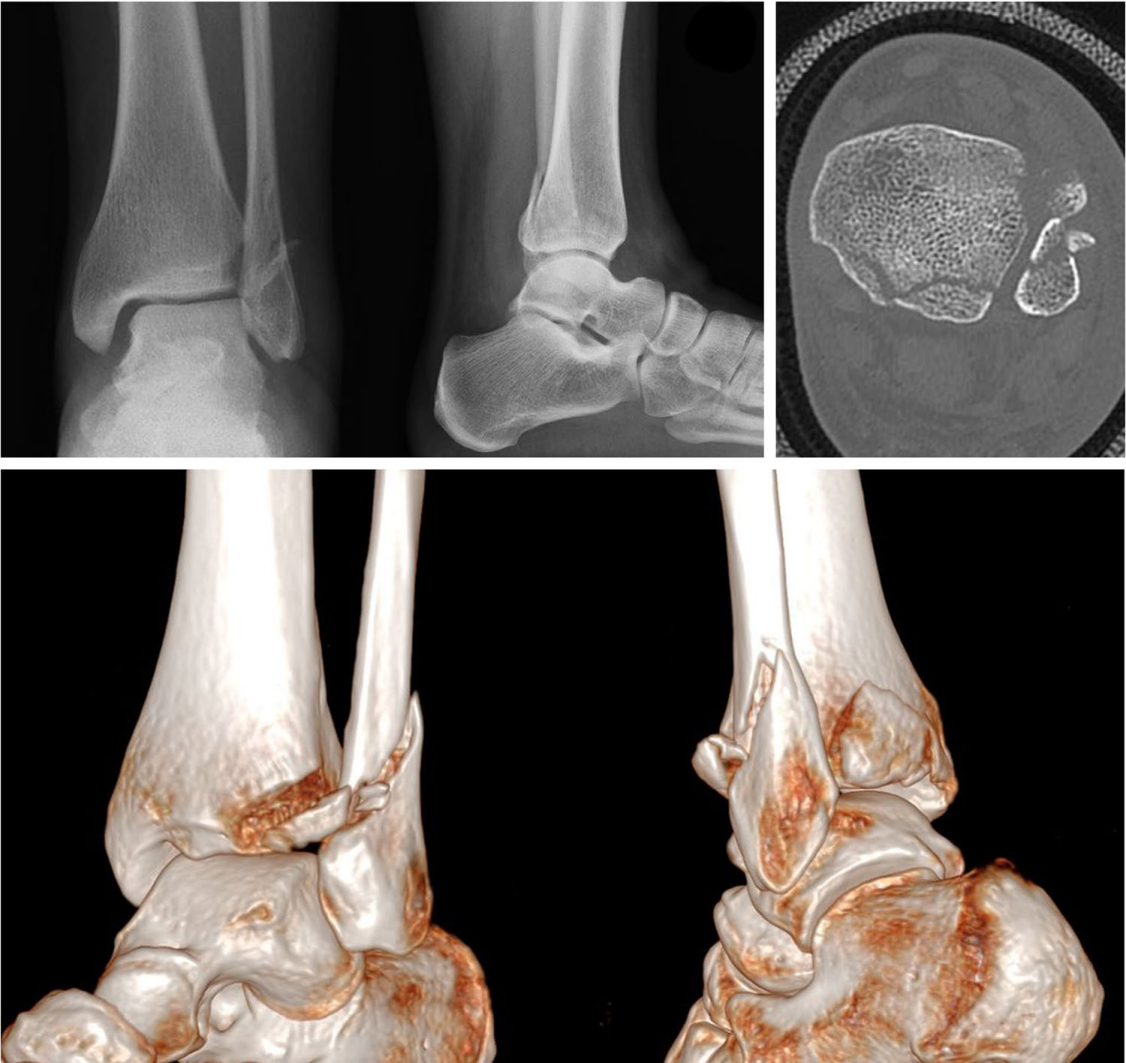
Standard X-rays and CT-scan showing a short oblique lateral malleolus fracture with anterior comminution, an avulsion fracture of the Chaput tubercle, and a displaced and comminuted fracture of the posterior malleolus Haraguchi 1/Bartonìček 2 (combination C)

As an illustrative case, we present a 35-year-old woman who sustained a Lauge-Hansen supination-external rotation stage IV of her left ankle while hiking. X-rays and CT-scan (Fig. 3) showed an ankle fracture with a comminuted Weber B-type fracture of the fibula, associated with an avulsion of the Chaput tubercle and a posterior malleolus fracture Haraguchi 1/Bartonìček 2.

The surgical procedure was performed 13 days after the injury. Approach was done as described in the “Surgical procedure” section above (Fig. 1a–c). In this case, the fracture of the lateral malleolus was reduced and fixed first, followed by the fixation of the posterior malleolus (Fig. 1e, f). Finally, the avulsed Chaput tubercle was fixed with a 3.5 mm screw (Fig. 1d).

**Fig. 4:**
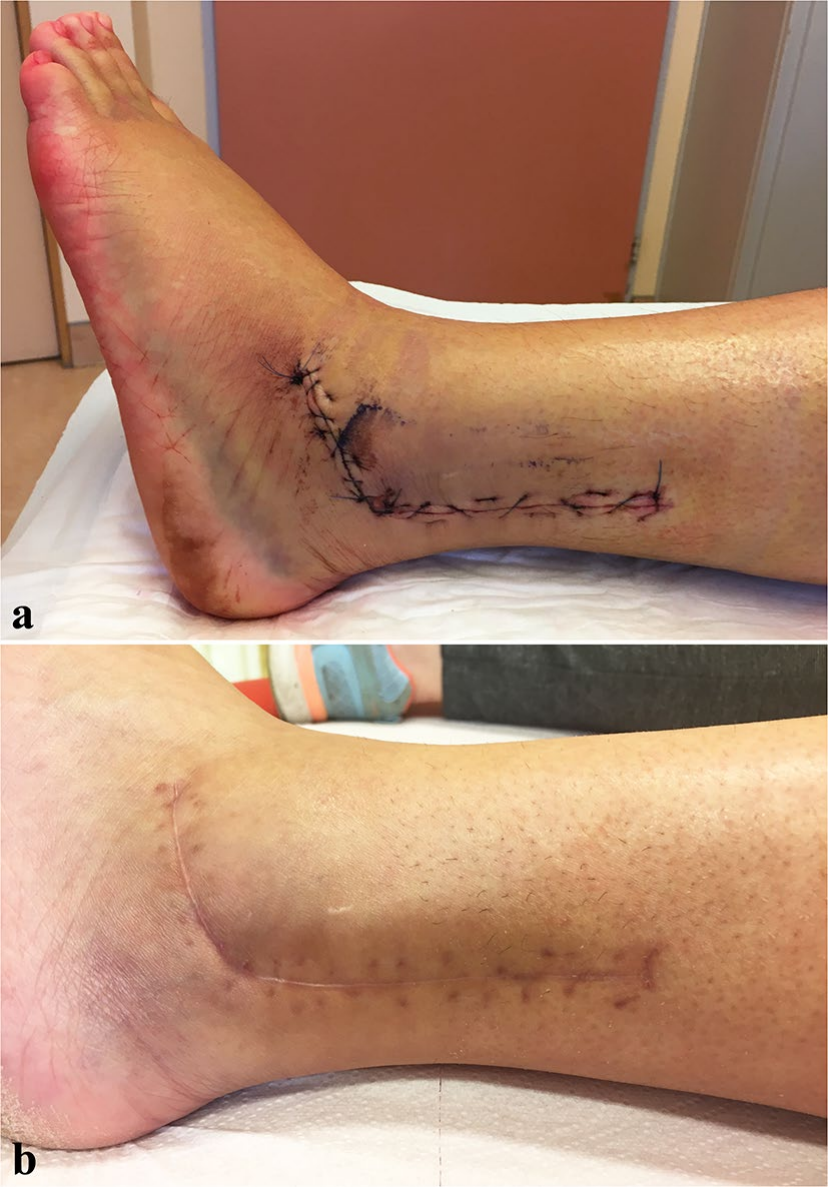
Scar at 4 postoperative days (**a**) and at 2 months (**b**)

Postoperative care was conducted as described above. Wound healing was achieved without complications (Fig. 4).

**Fig. 5:**
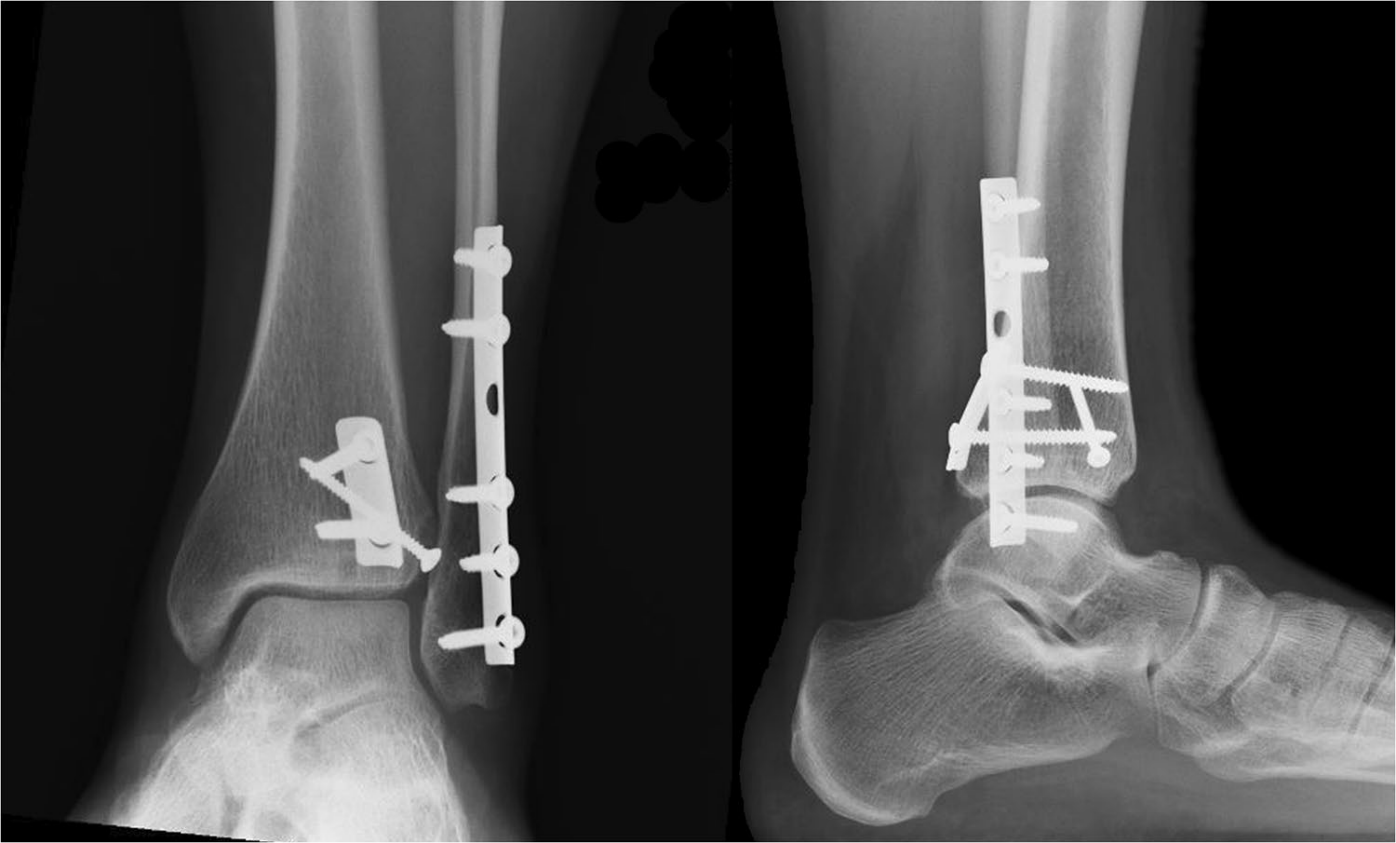
Weight bearing X-rays at 1 year

The postoperative X-rays showed anatomical reduction, as well as the control at 1 year (Fig. 5).

## Discussion

As the PAMELA involves a large cutaneous flap, the main potential concern of this novel approach was the wound healing. In this study, three patients developed delayed wound healing, which corresponds to 15% of the studied population. However, these cutaneous complications healed with local treatment, without the need of a revision surgery nor skin graft or flap. No infection occurred. The reduction was anatomical in all cases and no delayed complication occurred in our cohort followed-up for a median of 21 weeks. The only nerve complication was transient paresthesia in the sural nerve territory, with spontaneous recovery. Therefore, our study confirms the safety of this new extensive approach to address complex ankle fractures and highlights the advantages that it provides for anatomical reduction, which is known as an important factor in the functional outcome [1, 13].

The rate of wound complications is in line with early results and wound healing complications reported following surgical treatment of ankle fractures [14, 15]. Saleh et al. demonstrated 11.6% wound-healing complications in their studied population of 215 patients, half of which required operative interventions [14]. Cammas et al. [15] described 10% of wound complications in 433 patients; among patients with skin necrosis, 83% required surgical washing or debridement and 50% required local flap or skin graft coverage. Both above-mentioned studies showed that cutaneous complications increase with the severity of the fracture. In our cohort, all fracture patterns are severe, since the PAMELA is indicated only in case of complex fractures, as described in Table 1. Therefore, an increased risk of skin complications related to the complexity of the fracture was pre-existing in our patients, regardless of the approach chosen. Our results showed that wound healing complications occurred in the longitudinal part of the incision, and not on the cutaneous flap, and could, therefore, have occurred as well with a standard posterolateral incision. It should also be noted that the three delayed wound healing were only superficial. As the PAMELA is a novel approach, all patients were followed very closely, and any deviation from the standard healing process was recorded. Similar minor complications could not have been recorded in studies with retrospective design as mentioned above.

In the retrospective analysis of Cammas et al., 8% of patients had a postoperative unsatisfactory reduction based on standard X-rays [15]. Roberts et al. described a mean rate of 34% of malreduction in 261 ankle fractures, which increases with the severity of the fracture, from 15% for type B1 to 42% for type C [13]. This study further showed that the quality of reduction is correlated with functional outcomes [13], and that patterns of fracture including a fracture of the posterior malleolus lead to worse outcomes, even when reduced well. However, Jeyaseelan et al. [16] compared outcomes between fixed and unfixed posterior malleolus in 320 patients and demonstrated that fixation of posterior malleolus fractures lead to better functional score. Furthermore, Verhage et al. [2] showed that persistent intraarticular step-off of posterior malleolar fragment was a risk factor for development of osteoarthritis after a mean follow-up of 6 years. The importance of an anatomical reduction of the posterior malleolus in the clinical outcomes was also demonstrated in several other studies [17–19]. Moreover, the fixation of the posterior malleolus reduced the need for trans-syndesmotic screw [16, 18]. Thus, even if the fixation of posterior malleolar fragment was controverted in the past, it is now well recognized, regardless of the size of the fragment.

The first classification of the posterior malleolus fracture, based on axial CT-scan slices, was proposed by Haraguchi et al. [10]. Then, Bartonìček et al. [11] described a novel classification based on CT-scan 2D and 3D reconstruction. According to Bartonìček, the type 2 is the more frequent, followed by type 3, 4 and 1. We found similar results in our cohort. Although we proposed, in our previous publication [9], to consider performing the PAMELA only for Bartonìček type 2 and 4, our clinical practice has shown that some Bartonìček type 3, those with a simple undisplaced medial fragment, can also be treated through a posterolateral approach, as we treated seven patients with optimal results. This is also advocated by Bartonìček et al. [20], who proposed posteromedial approach for type 3 fractures only if the posteromedial fragment cannot be visualized and fixed through the posterolateral approach.

This study as several limitations. First, the population was small, which does not allow to generalize the results and the complication rate. Only one patient was an active smoker, two were diabetics, and the oldest patient was 73. Thus, we could not make any assertions about the cutaneous healing in these populations. Second, this is a retrospective study. However, the data have been collected prospectively, making the assessment of the results reliable. Finally, the reduction of the fracture was only assessed with standard X-rays. To ensure perfect anatomical reduction, CT-scan would be required. Nevertheless, we think that more irradiation is not necessary, because it is not associated with any benefit for the patient if the clinical course is satisfactory.

Despite these limitations, the present study shows encouraging results with optimal wound healing in the majority of the patients in the early postoperative period, and complete healing in all patients at 8 postoperative weeks without the need for revision surgery. Furthermore, the PAMELA permits to obtain anatomical reduction in all patients, and some Bartonìček type 3 posterior malleolar fractures with a simple medial extending configuration can also be addressed with this approach.

## Conclusion

The major interest of this novel approach is to make possible to address fractures of both the posterior and lateral malleoli and of the anterolateral portion of the ankle, through a single incision. The PAMELA offers, therefore, new perspectives in the management of complex ankle fractures. A larger prospective study is ongoing in our institution to confirm our present results.
